# Early Detection of NSCLC with scFv Selected against IgM Autoantibody

**DOI:** 10.1371/journal.pone.0060934

**Published:** 2013-04-09

**Authors:** Tetyana Pedchenko, Ray Mernaugh, Dipti Parekh, Ming Li, Pierre P. Massion

**Affiliations:** 1 Department of Medicine, Vanderbilt Ingram Comprehensive Cancer Center, Vanderbilt University School of Medicine, Nashville, Tennessee, United States of America; 2 Department of Biochemistry, Vanderbilt University School of Medicine, Nashville, Tennessee, United States of America; 3 Department of Biostatistics, Vanderbilt University School of Medicine, Nashville, Tennessee, United States of America; 4 Veterans Affairs, Tennessee Valley Health Care Systems, Nashville, Tennessee, United States of America; University of Pittsburgh, United States of America

## Abstract

Survival of patients with lung cancer could be significantly prolonged should the disease be diagnosed early. Growing evidence indicates that the immune response in the form of autoantibodies to developing cancer is present before clinical presentation. We used a phage-displayed antibody library to select for recombinant scFvs that specifically bind to lung cancer-associated IgM autoantibodies. We selected for scFv recombinant antibodies reactive with circulating IgM autoantibodies found in the serum of patients with early stage lung adenocarcinoma but not matched controls. Discriminatory performance of 6 selected scFvs was validated in an independent set of serum from stage 1 adenocarcinoma and matching control groups using two independent novel methods developed for this application. The panel of 6 selected scFvs predicted cancer based on seroreactivity value with sensitivity of 0.8 and specificity of 0.87. Receiver Operative Characteristic curve (ROC) for combined 6 scFv has an AUC of 0.88 (95%CI, 0.76–1.0) as determined by fluorometric microvolume assay technology (FMAT) The ROC curve generated using a homogeneous bridging Mesa Scale Discovery (MSD) assay had an AUC of 0.72 (95% CI, 0.59–0.85). The panel of all 6 antibodies demonstrated better discriminative power than any single scFv alone. The scFv panel also demonstrated the association between a high score - based on seroreactivity - with poor survival. Selected scFvs were able to recognize lung cancer associated IgM autoantibodies in patient serum as early as 21 months before the clinical presentation of disease. The panel of antibodies discovered represents a potential unique non-invasive molecular tool to detect an immune response specific to lung adenocarcinoma at an early stage of disease.

## Introduction

Non-small cell lung cancer (NSCLC) is the most prevalent form of lung cancer and, if not detected early, has a low cure rate. Survival rate improves dramatically from 13% when diagnosed at stage IIIA to 70% when detected at stage IA [1]. Currently only 16% of patients are diagnosed at stage I [2] Early detection of lung cancer represents a critical and challenging need in the management of this deadly disease. Despite recent advances in molecular diagnostics, no specific biomarker for the early detection of lung cancer has reached the clinic.

A promising approach to the early detection of cancer is to look for the immune response to the developing cancer. Solid tumors produce unique tumor associated antigens (TAA) that are recognized by the immune system resulting in the production of autoantibodies against them. Growing evidence indicates that humoral immune response in the form of autoantibodies is present in cancer patients before clinical demonstration of disease [3], [4]. It has been reported that cancer-associated autoantibodies can be detected up to 5 years before symptomatic disease [5]. As cancer develops, antibodies can be produced against proteins that are up-regulated or modified in the cancerous cells. For example, in a study of lung cancer, sera from 60% of patients with lung adenocarcinoma and 33% of patients with squamous-cell lung carcinoma, contained immunoglobulins that interacted with glycosylated annexins I and II, while sera from non-lung cancer patients did not [6]. A number of tumor-associated antibodies have also been detected by screening expression libraries with patient sera [7]–[10] or, more recently, by using a random peptide-library approach [11]. Yet, despite recent advance, current research has not achieved the final clinical goal of detecting biomarkers specific for the early detection of lung cancer.

Antibodies represent some of the most abundant proteins in human serum and can be present in serum at concentrations that supersedes that of their respective antigens. The amino acids present in an antibody’s heavy and light chain variable (V-region) or idiotypic regions interact with and bind to an antigenic site, and are unique to the antibody. Antibodies known as anti-idiotypic antibodies can, like an antigen, also interact with some or all of the same amino acids that make up the idiotype of an antibody.

IgM antibodies are the first class of antibodies produced during a humoral immune response, and represent the most logical class of antibodies to detect as early indicators of disease. Antibody class switch from IgM to IgA, IgE or IgG is dependent upon antigen presentation by MHC molecules present on T-cells and the antibody producing B-cells. Typically, MHC molecules present peptide fragments, but do not usually present carbohydrate or other non-amino acid fragments. If an IgM antibody is produced to a site on a self- or “cancer antigen” that has been glycosylated in response to tumor formation, then it is unlikely that other classes of antibodies will be made to the exact same site if MHC presentation does not occur. We have tested the hypothesis that IgM antibodies produced against proteins up-regulated or modified in response to tumor formation would be present and more readily detected than their respective antigens; and, as such, would represent useful biomarkers as indicators of early stage lung cancer.

The goal of this study was to use phage-display to select for single chain fragment variable (scFv) antibodies for use in assays to detect cancer-associated IgM autoantibodies as biomarkers of early stage NSCLC. Since antibodies of identical antigen-binding site specificities have similar or identical idiotypic amino acid sequences, then any IgM antibodies that undergo IgM to IgA, IgE or IgG class switch - without undergoing significant amino acid change due to V-region somatic mutations - would also be detected by the same scFv, if the scfv were specific for the idiotype of an antibody.Antibody fragments, as Fab or scFv, have been among the first proteins to be displayed on the surface of a filamentous virus or bacteriophage using a procedure initially described in 1990 by McCafferty et al. [12]. Phage-display represents the most commonly used *in vitro* method to select for peptides and antibodies to target molecules [13].

Recognizing that the heterogeneity of NSCLC may determine the diversity of disease-related humoral immune response, we have restricted ourselves to the discovery of autoantibody present in adenocarcinoma - the most common type of lung cancer. To select for scFv binding to cancer patient IgM that could be early disease biomarker, sera from only stage I adenocarcinoma patients were used.

## Results

### Phage Selections and Assays for scFv Specific for Adenocarcinoma IgM Autoantibodies

**Figure 1:**
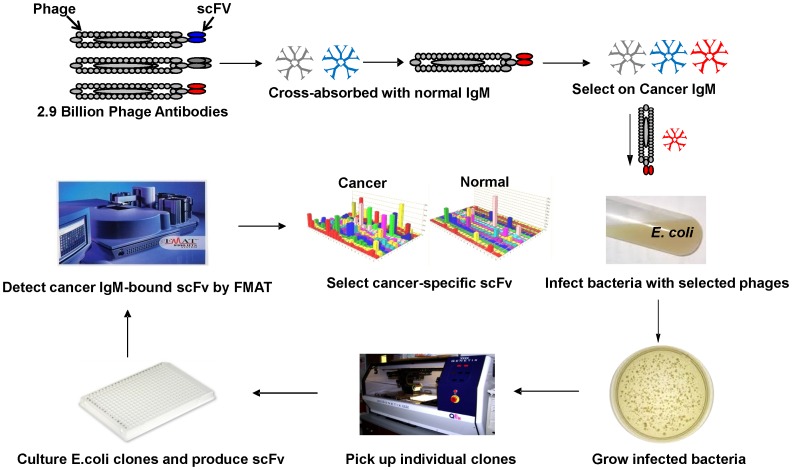
Procedure used to select cancer-specific recombinant scFv antibodies. A phage-displayed antibody library was applied to immobilized IgM obtained from pooled age, smoking history matched control samples, to remove phage-displayed scFv reactive with IgM present in both controls and patients with lung cancer. The cross-absorbed phage-displayed antibody library was applied to immobilized IgM purified from pooled lung cancer patients to obtain scFv specific for lung cancer IgM. Phage-displayed antibodies bound to immobilized IgM obtained from pooled lung cancer patients were eluted and used to infect *E. coli* and plated onto selective agar plates. Bacterial colonies from two rounds of selection were picked using a colony picker from agar plates to 384 well microtiter culture plates and induced to express soluble scFv. ScFv were transferred from 384 well culture plates to separate 384 well assay plates coated with lung cancer or control antibodies. Bacterial colonies producing scFv reactive in a homogenous fluorescent assay with lung cancer but not control antibodies were identified and used to produce larger quantities of scFv for use in subsequent assays.

**Figure 2:**
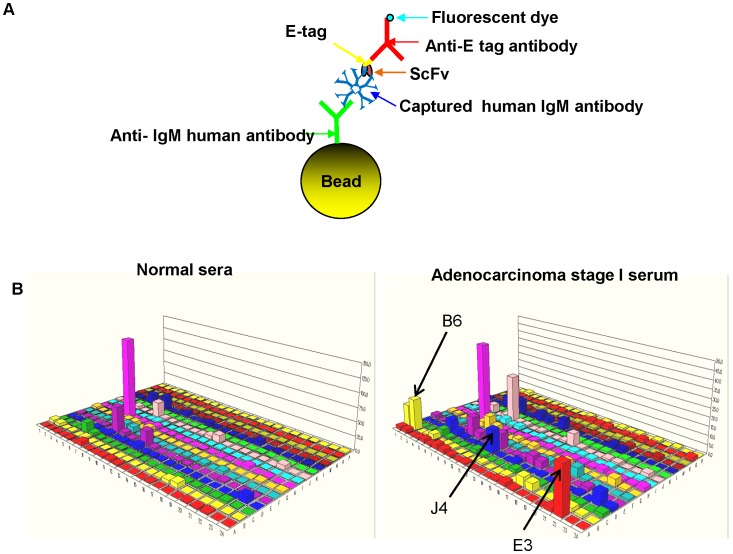
Approach used to determine scFv binding activity to serum IgM using fluorescent (FMAT) assay. **A.** Anti-human IgM - specific antibodies immobilized onto beads were used to capture IgM from human serum samples. Recombinant scFv antibodies bound to human IgM were detected with fluorescent-labeled secondary antibodies (specific for the E- tag on the scFv) using a fluorescent (FMAT8100) plate reader. **B.** Binding activity of 384 scFv to control or cancer serum as determined using Fluorometric Microvolume Assay Technology (FMAT). Each bar represents the fluorescent signal obtained when scFv bound to beads bearing human serum IgM. ScFv demonstrating differential binding to IgM from cancer and control serum samples were selected for the further analysis (see arrows indicating the signals for scFv selected in this representative experiment: B6, J4 and E3).

A phage-displayed scFv antibody library was used to select scFv specific for lung cancer IgM autoantibodies as biomakers of early stage I adenocarcinoma. The procedure is summarized in Figure 1 and is described in the Materials and Methods section. The phage antibody library was cross absorbed against IgM obtained from normal human serum samples to remove scFv cross-reactive with IgM from normal and adenocarcinoma serum samples. Phage scFv remaining after cross absorption were panned against IgM isolated from early stage I adenocarcinoma lung cancer patients to obtain scFv specific for lung cancer autoantibodies. Colonies obtained after two rounds of phage scFv cross absorption and selections were picked and induced to express scFv. Expressed scFv were initially analyzed using a FMAT homogenous assay and an FMAT 8100 plate reader (Figure 2A) to determine binding activity for IgM from individual early stage I lung cancer (positive control) patients and pooled normal (negative control) human serum samples. A total of 384 scFv (Figure 2B) identified in initial assays were re-assayed against IgM obtained from a pool of 8 early stage I adenocarcinoma patients and IgM obtained from a pool of 8 normal human serum samples to identify 9 scFv that preferentially bound IgM from early stage I lung cancer patients in comparison to normal patients (with p<0.05) as it is shown in Figure 2B for three scFvs – B6, J4 and E3,. To demonstrate that the observed differences are not due to the difference in IgM level in cancer versus non-cancerous serum samples, we performed IgM-specific ELISA on these serum samples. The IgM levels were not significantly different between two groups: optical density was 0.41±0.13 in cancer group and 0.37±0.18 in controls (p = 0.5) Three of the 9 bacterial clones producing scFv were not stable, did not produce enough scFv and were not used for subsequent assays.

### Diagnostic Performance of Selected scFvs in an Independent Sample Set

**Figure 3:**
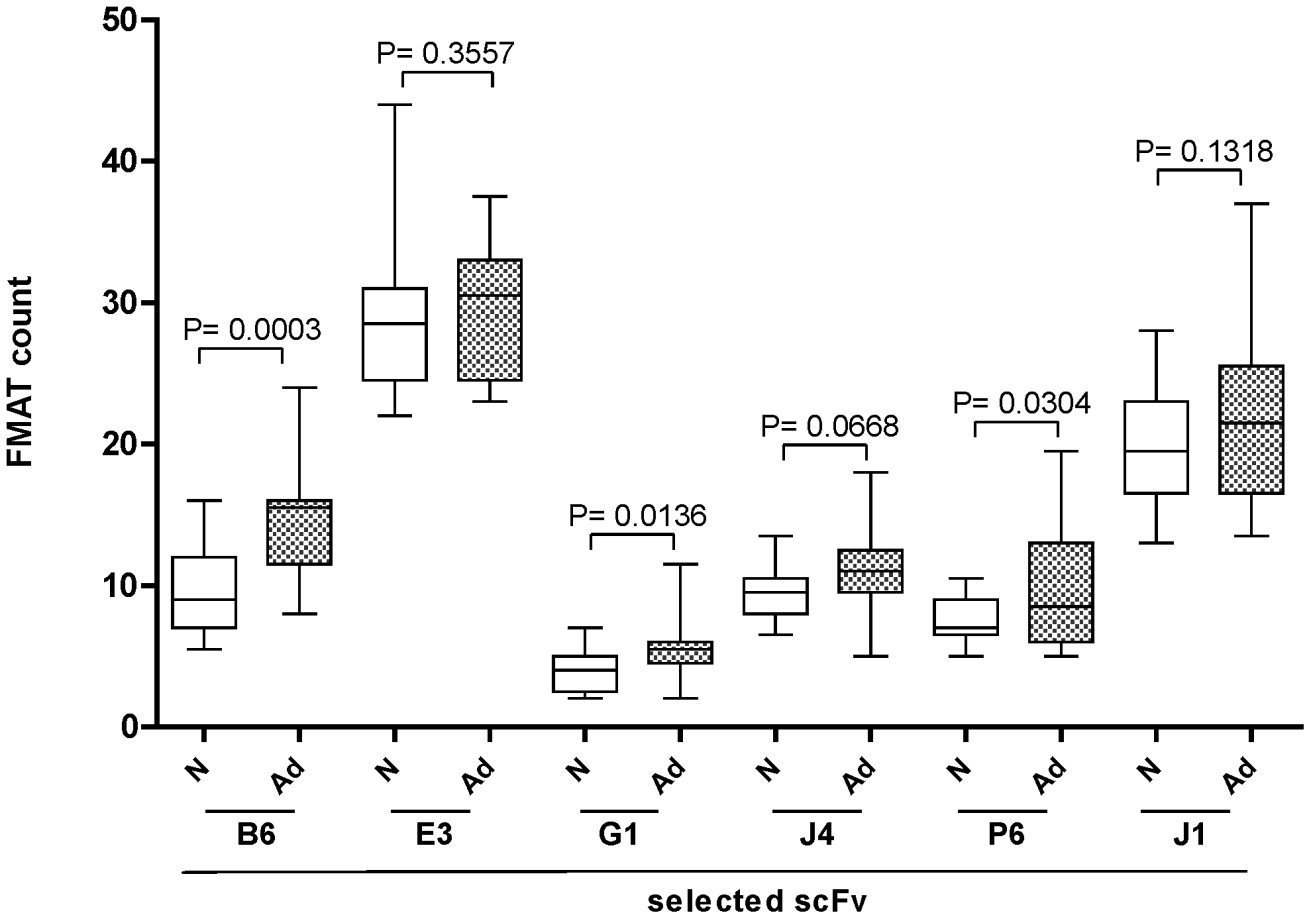
Panel of six scFv antibodies discriminated NSCLC from control serum in independent validation set. ScFv binding activity was determined by a fluorescent (FMAT) assay. Each bar represents the mean fluorescent intensity (FMAT count) of scFv with 15 non-canceorus control (N) or 15 adenocarcinoma stage 1 (Ad1). P-values are presented from two-tailed student t-test for each scFv (N vs Ad1 comparison).

**Figure 4:**
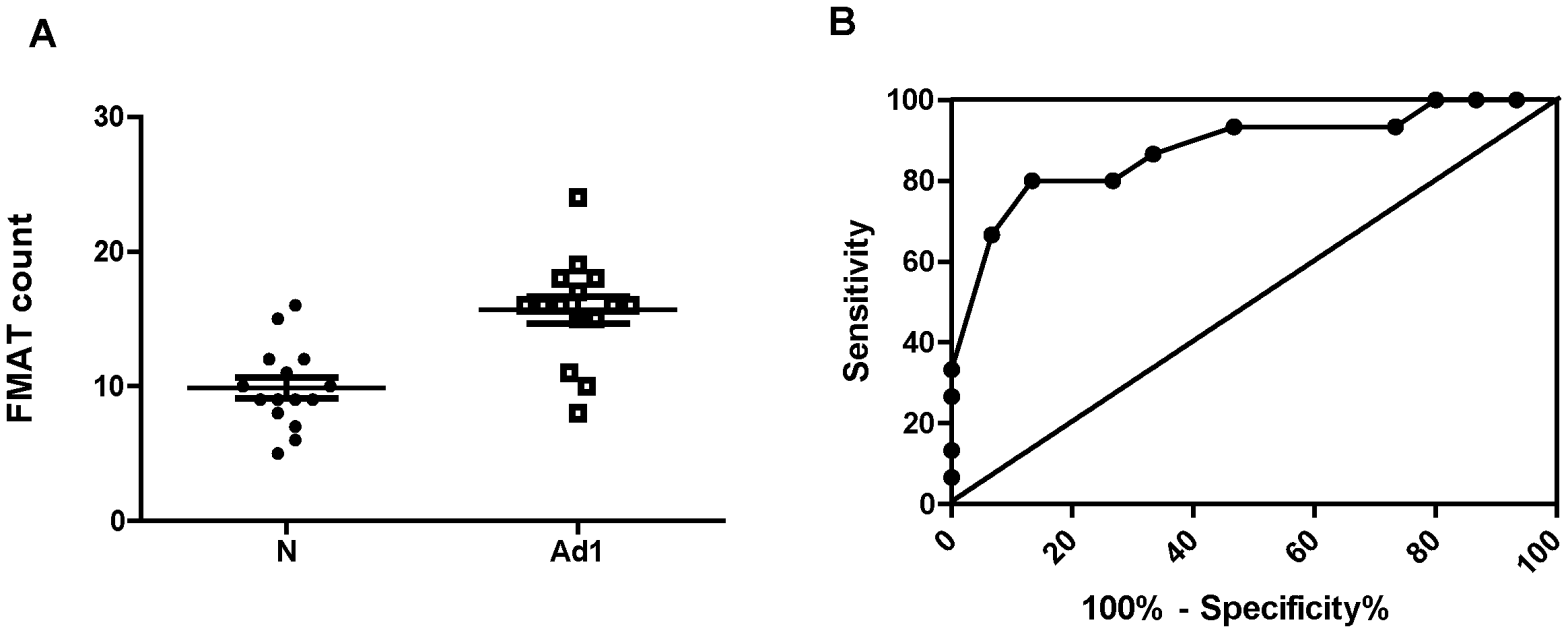
Discriminatory performance of single scFv in a fluorescent (FMAT) assay. **A.** ScFv B6 binding activity with individual serum samples from either non-cancerous (N) or stage 1 adenocarcinoma (Ad1) patients. Each dot represents the fluorescent assay signal intensity (FMAT count) for scFv with individual serum samples (n = 15N +15Ad1). **B.** A receiver operating characteristic (ROC) curve was generated for scFv clone B6 based on fluorescent assay signal intensity for 15 control and 15 adenocarcinoma stage 1 serum samples.

The 6 remaining scFv were further characterized by FMAT analysis using an independent set of 15 lung cancer and 15 non-cancer serum samples. Three of the 6 scfv (designated B6, G1 and P6) showed significant difference between cancer and negative control groups (Figure 3). The scFv designated as B6 demonstrated the best performance with an AUC of 0.84 (95% CI, 0.71–0.97) (Figure 4). Receiver operating characteristic (ROC) curves with corresponding area under the curve (AUC) values were generated for all six scFv based on their seroreactivity (Table 1). The 6 scFv were then evaluated as components of a panel of scFv to determine if the scFv panel could discriminate patients with adenocarcinoma stage I from non-lung cancer patients. In FMAT assays, the scFv panel discriminated lung cancer patients from non-lung cancer humans with a sensitivity 0.8 and a specificity 0.87 (Table 1). The ROC for the scFv panel had an AUC of 0.88 (95%CI, 0.76–1.0) with the scFv panel demonstrating better discriminative power than any single scFv. We recognize that our sample size (30 patients) may be not sufficient to make a stable prediction for the 6 antibodies tested simultaneously. In addition to backward selection method for variable reduction, we also provided a compound score method by summarizing all 6 scFv as one individual risk factor in the logistic model. A bias-correct AUC of 0.78 was obtained by bootstrap validation model [14].

**Table 1:** Prediction of cancer *versus* non-cancerous based on scFv seroreactivity with independent set in FMAT assay (n = 15N+15Ad).

scFV ID	Specificity	Sensitivity	Overall	ROC
B6	0.80	0.67	0.73	0.84
3E	0.53	0.60	0.57	0.57
G1	0.67	0.73	0.70	0.47
J4	0.73	0.60	0.67	0.64
P6	0.73	0.60	0.67	0.69
J1	0.60	0.60	0.60	0.59
All three(B6,G1,P6)	0.87	0.67	0.77	0.87
**All six**	**0.87**	**0.80**	**0.83**	**0.88**

### A Novel scFv-based Electrochemiluminescent Assay to Detect Lung Cancer Autoantibodies

**Figure 5:**
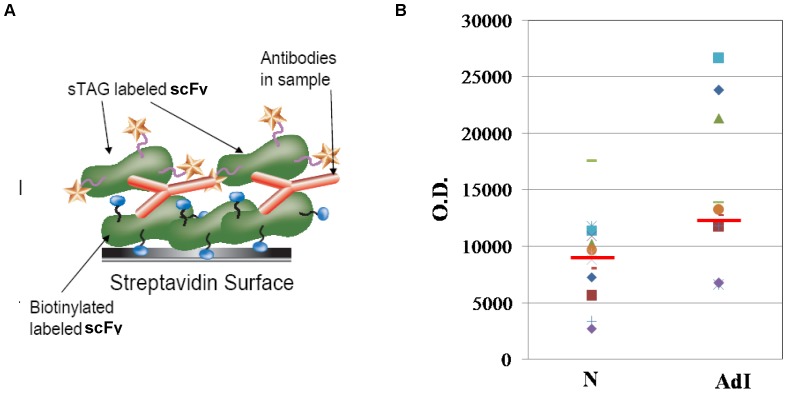
ScFv binding activity as determined using a novel homogeneous bridging electrochemiluminescent (MSD) assay. **A** Biotinylated scFv immobilized onto streptavidin-coated microtiter wells were used to capture autoantibodies. Captured autoantibodies were detected using Sulo-Tag labeled scFv and an MSD plate reader. **B** Binding activity of scFv antibody with autoantibodies obtained from stage I lung adenocarcinoma (AdI, n = 14) and from non-cancer (C, n = 21) human serum samples. Each data point represents the assay signal intensity for individual serum samples. P = 0.031 for normal (C) vs cancer (AdI) group average (horizontal lines).

Each IgA, IgG and IgE antibody has, at minimum, 2 identical sets of idiotypes or heavy and light chains, while IgM antibodies have 10. Therefore, mono-specific scFv stemming from the same bacterial clone can bind, at minimum, 2 sites on the same “autoantibody”. As such, a mono-specific, monoclonal scFv can conceivably be used in the same assay to both capture and detect the same “autoantibody” to enhance assay specificity. We developed and utilized an electrochemiluminescent homogenous assay to confirm results obtained using the FMAT homogenous assay. An aliquot of a single scFv (e.g. B6) was biotinylated and used to capture autoantibodies from serum samples. Another aliquot of the same scFv (i.e. B6) was conjugated with an MSD sulfo-Tag and used to detect autoantibodies captured by the biotinylated (i.e. B6) scFv (see Figure 5A). Homogenous bridging electrochemiluminescent assay results were then obtained using a Mesa Scale Discovery (MSD) plate reader [14]. The assay using the B6 scFv was able to discriminate patients with stage I adenocarcinoma from normal matched controls (Figure 5B). Three samples from patients with squamous carcinoma and one sample from a patient with broncho-alveolar carcinoma that were included in the set failed to demonstrate serological activity with the B6 scFv (data not shown). Serum samples diluted up to 600 fold were useful in assays incorporating any of the scFv. However, for the B6 scFv, some serum samples could be diluted 3–5,000 fold (data not shown).

### Validation of Selected scFvs in Independent Serum Sample Set Using Novel MSD Assay

**Figure 6:**
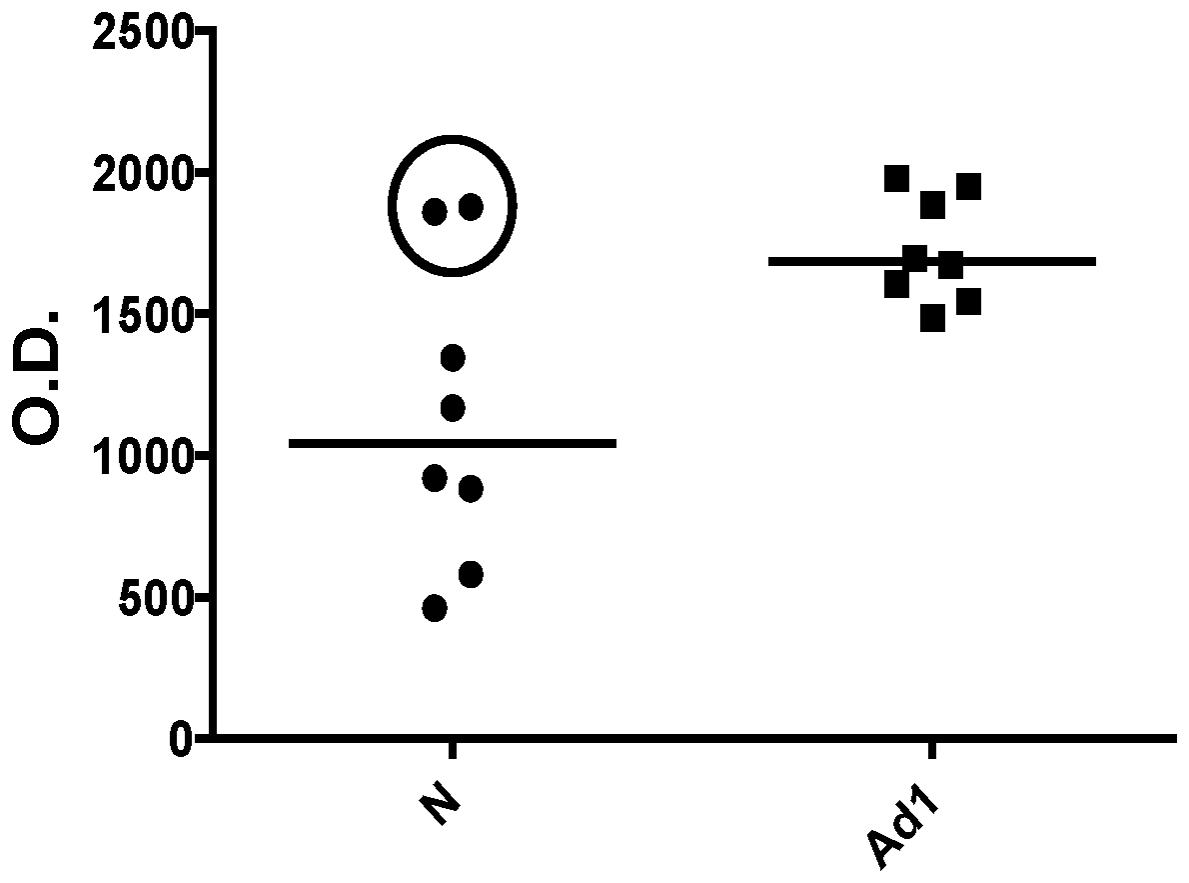
Binding activity of scFv J4 on human serum samples in homogeneous bridging electrochemiluminescent (MSD) assay. Data points represent binding activity for scFv J4 on individual serum samples. Two outliers in control group that demonstrated high binding activity are circled. C - non-cancerous control sample, Ad1-stage 1 adenocarcinoma.

For validation of 6 scFvs in homogenous bridging assay all 43 samples were used. The discriminatory capacity of selected scFvs was evaluated for individual clones as well as for their combination. Validation was done with the independent set of 43 samples: 22 early adenocarcinoma samples (19 of stage I and 3 of stage IIB), and 21 matched controls (Table 2). These 2 groups were matched by age, gender and smoking history. The control group included patients with different lung inflammatory diseases including COPD, asthma and granulomatous disease. The generated ROC curve had AUC 0.72 (95% CI, 0.59–0.85) (Table 3). There were 4 serum samples from control group that demonstrated highest immunoreactivity among non-cancerous samples with 4 out of 6 tested scFv recombinant antibodies designated as J4, G1, P6 and B6. Example of two serum samples that demonstrated highest titer of autoantibody reactive with scFv clone J4 in non-cancerous group is shown in Figure 6 (circled). All samples used in this study was collected and banked during years of 2005–2008. Follow-up on these patients demonstrated that 2 patient from control group with elevated autoantibody titer have developed lung cancer later on. Patient #1 was diagnosed with lung cancer 18 months after the blood was collected and patient #2 was diagnosed with stage IV NSCLC 21 months after blood collection. Patient #3 had COPD and a suspicious lung nodule and died 22 months later of heart failure. Patient #4 had COPD, lung nodules, granulomatous inflammation and atypical mycobacterium infection at the time of blood collection and died 2 years later. None of the other control subjects demonstrated elevated autoantibody titers, and all are still alive and disease free 4 years later. We did not exclude 2 patients that developed cancer later on from control group in our analysis.

**Table 2:** Patients and samples clinical characteristics.

	Control	NSCLC	Total
**Sample size**	21	22	43
**Gender:**			
Female	10	10	20
Male	11	12	23
**Smoking status:**			
Never	5	1	6
Ex-smoker	8	20	28
Current	8	1	9
**Smoked packs per year**	43.4 (0–165)	51.7 (0–150)	n/a
**Age at blood collection and/or diagnosis**	67.5 (43.0–87.0)	64.2 (47.0–85.0)	n/a
**Tumor pathological staging**:			
I	n/a	1	1
IA		11	11
IB		7	7
IIB		3	3

**Table 3:** Prediction of cancer *versus* non-cancerous based on scFv seroreactivity with independent set in MSD assay (n = 22N+21Ad).

scFV ID	Specificity	Sensitivity	Overall	ROC
B6	0.57	0.57	0.57	0.58
3E	0.62	0.30	0.45	0.53
G1	0.62	0.61	0.61	0.65
J4	0.33	0.57	0.45	0.47
P6	0.43	0.43	0.43	0.47
J1	0.57	0.35	0.45	0.45
**All six**	**0.71**	**0.61**	**0.66**	**0.72**

**Figure 7:**
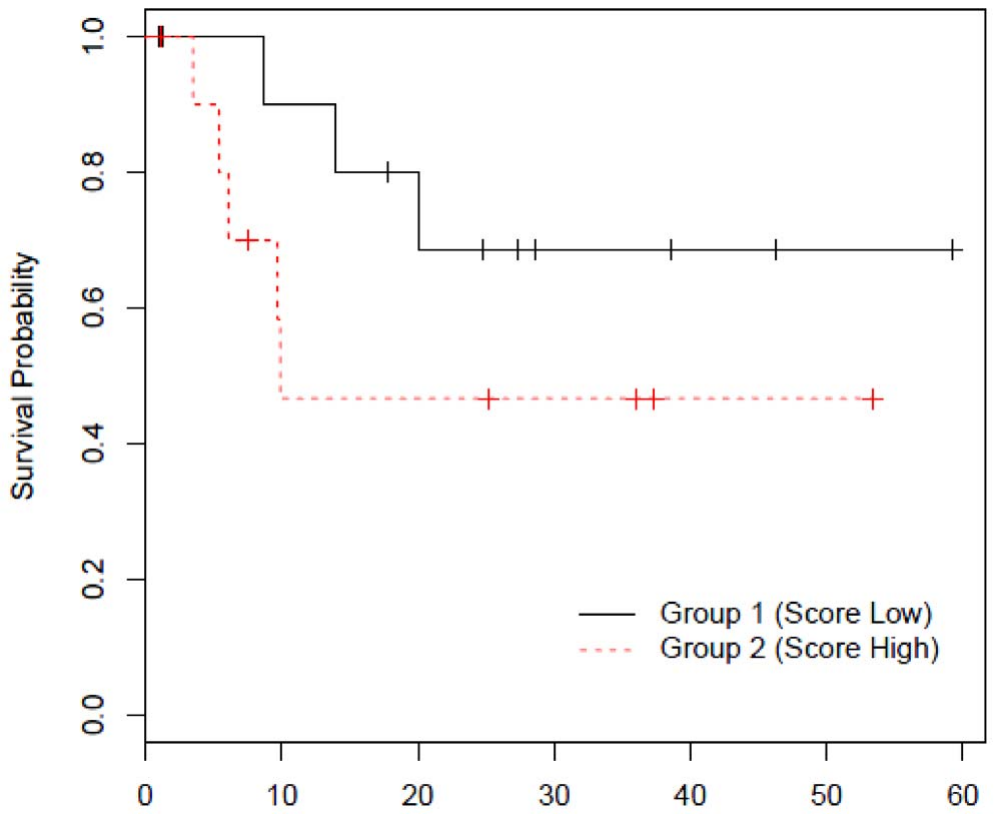
Panel of 6 scFv demonstrated an association between assay signal intensity and survival. The prediction model presented uses assay signal intensity to generate low/high score. High score (dotted line) is associated with a lower probability of survival.

Although survival was not our primary outcome, we speculated based on our observation that seroreactivity of selected scFvs could be associated with prognosis. We used the panel of all 6 scFv to build a survival prediction model to compare the equality of survival curves for the low and high scFv score groups (the score was generated using a linear combination of 6 individual scFv signal intensity). The panel demonstrated the associating of high score with poor survival (Figure 7). However, log-rank test shows that it is not statistically significant.

## Discussion

We took advantage of the exquisite sensitivity of the immune system to detect lung cancer specific antibodies at an early stage in tumor progression and develop a new autoantibody testing platform. Autoantibody to tumor-associated antigens was found in the circulation of patients in the preclinical setting [5]. It can be detected and measured in simple immunoassay and their presence or concentration can be correlated with occurrence of disease. As such, the autoantibody can serve as biomarkers for early diagnosis. Autoantibodies represent attractive candidates as a class of biomarkers also due to their robustness, stability and extensive set of tools available for their detection and quantification.

We decided to focus this research project on stage 1 NSCLCs because prompt management of malignant pulmonary nodules presenting at stage I (T1, N0, M0) could have 5 year survival rate up to 80%, and because NSCLC is the most prevalent in heterogeneous populations of lung cancer.

We used large phage-display recombinant scFv antibody library to select for lung cancer specific circulating autoantibodies. There are several advantages in using this approach: 1) the binding strength of the phage-displayed recombinant antibodies due to their larger size is greater than that for a phage-displayed peptide, resulting in better specificity, selectivity and potency of antigen binding; 2) the ability to use the E-tagged scFv antibodies to affinity-purify and conjugate lung cancer-specific antibody/antigen for subsequent characterization; 3) the affinity-purified recombinant antibodies can be labeled with biotin, dyes, enzymes, or conjugated to beads, and be used in a variety of inexpensive immunoassay formats.

We successfully selected for scFv recombinant antibodies reactive with circulating IgM autoantibodies obtained from adenocarcinoma patient but not normal human samples. In a validation set of patients matched by age, gender and smoking status, 6 scFv discriminated serum samples from stage I adenocarcinoma and no-cancer control at very significant statistical level.

A novel homogeneous bridging scFv assay was developed using MSD technology. This technology has demonstrated more sensitivity and broad dynamic range and it requires significantly less volume of serum sample than ELISA, which is commonly used in similar serological studies. The ability to multiplexing is also benefit over standard ELISA. In contrast to other multiplex platforms, such as Luminex, the MSD technology is advantageous because it does not require chemical conjugation of antigens to solid phase, therefore minimizing the potential impact on immunogenicity of tested antibody. This platform can be used in multi-array format for the high-throughput screening. To our best knowledge, we were the first to develop this type of assay using scFv for the autoantibody detection.

We were able to detect high titers of IgM lung cancer associated autoantibodies in the serum of patients 2 year before clinical presentation. This is what makes this approach promising. If such observation is confirmed, it would be of great interest to identify the proteins that changes so early at disease onset and these changes are so dramatic that organism starts to consider these proteins as “non-self” and develops autoantibody against them. We believe that these autoantibodies can be detected and measured in a simple immunoassay and their presence or concentration can be correlated with occurrence of disease.

The identification of panels of antibodies that are specific for particular cancer type may yield a molecular signature for non-invasive early diagnosis. While surgical resection is the most effective therapy in NSCLC, 75% of patients with lung cancer present with unresectable disease. A set of serological biomarkers may offer cost-effective ways for early detection of lung cancer, and by detecting NSCLC early in its course patients could be provided a better chance for a cure. Additional validation of our results is essential and would be particularly well suited in independent cohorts of high-risk individuals for lung cancer development. Upon follow up, we may be able to detect lung cancer earlier in patients found to have chest CT-detected lung nodules or ground glass opacities. Further investigations towards the identification of the targets may help us gain insight in the nature of the signal and the biology of the early disease process.

## Materials and Methods

### Ethics Statement

The Vanderbilt University and Nashville Veterans Affairs Medical Center Institutional Review Boards approved the study and all patients provided written informed consent. Copies of all written consent forms are available under IRB Protocol 030763.

### Serum Samples

Blood was collected from patients diagnosed with lung cancer and from non-cancerous patients. All cancer samples were collected prior to resection - at the time of diagnosis. Patients’ characteristics are presented in Table 2. All non-cancer and cancer patients used in this study were matched by age, gender and smoking status. The controls included patients with COPD and other lung diseases (e.g. mycobacterial infection) to account for immune response associated with inflammatory processes. Peripheral blood was collected and processed using a standard operating procedure [15]. Blood was drawn into a 7 ml serum vacutainer tube (Becton Dickinson, Franklin Lakes, NJ) without additive, incubated at room temperature for 30 min, centrifuged, aliquoted, snap frozen within 2 hours of collection and stored at −80°C until analysis.

### Phage-displayed scFvscFv Library

Modifications of previously published protocols [16] were used to develop a large (∼2.9×10^9^ or 2.9 billion members) phage-displayed ScFv (single-chain fragment variable) recombinant antibody library. The library has been used to obtain antibodies to different recombinantproteins [17], [18], [19], [17], [18], [19] hapten [20], peptide [10] and bacterial [21]protein antigens, metals [22]and an early breast cancer biomarker [23,23], with affinities as high as 65 pM. The scFv are encoded within the pCANTAB5E phagemid vector. The vector contains an ampicillin resistance gene to select for bacterial clones that contain the scFv-bearing phagemid. All scFv are expressed either as a phage gene 3 fusion protein (for phage display purposes) or as a ∼27 kD, soluble E-tagged scFv. The 11 amino acid E-tag sequence, present on each E-tagged scFv, is recognized by the Anti-E monoclonal antibody (G.E. Healthcare cat # 27-9412-01). The Anti-E monoclonal antibody is used in immunoassays to detect E-tagged scFv bound to antigens, or is used to affinity-purify E-tagged scFv produced in *E. coli*.

### Phage-displayed scFv Selection

Phage-displayed scFv specific for adenocarcinoma IgM were selected using IgM from human samples bound to beads. The following 11 steps were used to obtain scFv specific for andenocarcinoma IgM: 1) The phage-displayed scFv library was cross-absorbed against goat anti-human IgM (μ-chain) specific antibodies coupled to agarose beads (Sigma cat# A9935) to remove scFv reactive with goat antibodies. 2) Ten to 20 µl of serum from stage I adenocarcinoma patients was applied to 100 µl of goat anti-human IgM agarose beads to capture IgM from serum. Beads bearing human IgM were washed with PBS containing 0.1% Tween-20 (PBS-T) to remove non-bound human serum components. 3) The cross-absorbed phage-displayed scFv from step 1 was applied to beads from step 2 and incubated overnight at 4–8°C. Beads were washed with PBS-T to remove unbound phage-displayed antibodies, after which phage on beads were used to infect *E. coli* TG1 cells. 4) Infected TG1 cells were plated onto 2xYT agar plates containing ampicillin and 2% glucose (2xYTAG). Colonies stemming from this 1^st^ round of phage antibody selection were M13KO7 helper virus rescued to produce phage-displayed scFv and then PEG precipitated. 5) Human IgM antibodies pooled from 8 normal human serum samples were immobilized onto anti-human IgG agarose beads as described in step 2. 6) Beads from step bearing normal human IgM were used to cross-absorb PEG precipitated phage-displayed scFv to remove scFv cross-reactive with normal and adenocarcinoma IgM antibodies. 7) Nunc Maxisorb tubes were coated with goat anti-human IgM μ-chain specific antibody (Sigma cat#I0759) at 5 µg/ml in PBS and blocked with PBS-T. 8) Unbound phage-displayed scFv from step 6 were cross-absorbed against goat anti-human IgM in Maxisorb tubes (step 7) to remove scFv reactive with goat anti-human IgM antibodies.9) Eight Nunc Maxiasorb tubes coated with goat anti-human IgM antibodies were used to separately capture IgM from 10 µl of each of 8 different human adenocarcinoma serum samples. 10) Unbound phage-displayed scFv were applied to tubes from step 8 to select for scFv specific for adenocarcinoma IgM. 11) Bacterial colonies stemming from the 2^nd^ round of phage selection (step 10) were pooled and frozen down as glycerol stocks for use in subsequent assays to identify colonies producing scFv that bind to adenocarcinoma but not normal human IgM. It should be noted here that eight 1^st^ and 2^nd^ round phage selections were performed on 8 individual stage I adenocarcinoma serum samples, with all selections for each patient sample being kept separate.

### Bacterial Expression and Preparation of scFv in Periplasmic Extract

Cells from frozen glycerol stocks stemming from the 2^nd^ round phage scFv selections were plated onto 2xYTAG agar plates to obtain individual colonies. Individual colonies were picked from 2xYTAG agar plates into 384 well microtiter plates containing 2xYTAI (ampicillin and 1 mM IPTG) to induce *E. coli* scFv expression. A 384 well pin replicator was used to transfer bacteria from 384 well 2xYTAI to prepare 2xYTAG agar master replica plates. Cells in 2xYTAI microtiter plates were centrifuged to pellet cells. The supernatant was removed and cells were suspended in 40 µl of TES [0.2M Tris-HCl (pH8.0), 0.5 mM EDTA, 0.5M sucrose] and 60 µl of 1/5^th^ X TES (1 volume of TES plus 4 volumes of distilled water) then incubated on ice for at least 1 hour to obtain scFv in *E. coli* periplasmic extract.

### Anti-E Tag Monoclonal Antibody Conjugation to Alexa 647

The Anti-E tag monoclonal antibody conjugated to Alexa 647 was used for FMAT assays (Fluorometric Microvolume Assay Technology) to detect E-tagged scFv bound to adenocarcinoma IgM bound on beads. Briefly, the Anti-E tag monoclonal antibody was conjugated to Alexa 647 (Invitrogen cat# 20006) at a ratio of 2.25 dye molecules per antibody. Unincorporated dye was removed from the dye-labeled antibody using a NAP-10 column (G.E. Healthcare cat#17-0854-02) equilibrated with PBS.

### FMAT Assays to Identify scFv Specific for Adenocarcinoma IgM

Streptavidin-coated microtiter beads (Spherotech cat# SVP-100-4) at a concentration of 5 µg/µl were coupled with 0.2 µl biotinylated goat anti-human IgM (Sigma cat# B1265) per 1 µl of beads, washed with PBS-T to remove unbound anti-human IgM, then blocked with PBS-T. Approximately 0.6 µl of human serum was added per 1 µl of beads to immobilize normal or adenocarcinoma human serum IgM onto beads. Beads were washed with PBS-T to remove unbound IgM. Alexa 647 conjugated Anti-E tag monoclonal antibody was added to beads to a final concentration of 0.38 µg/ml of beads. Fifty microliters of beads containing Alexa 647 conjugated to Anti-E tag monoclonal antibody were dispensed to individual microtiter wells (∼0.138 µl of beads/well) of a 384 well Corning (cat#3912) microtiter assay plate. Fifteen µl of scFv in *E. coli* periplasmic extract were transferred from 384 well culture plates to corresponding wells in 384 well assay plates. Assay plates were incubated for 4 hours at room temperature, or overnight at 4–8°C in the dark. Assay plates were then read using an FMAT 8100 plate reader (Applied Biosystems).

### Homogeneous Bridging Assay on an MSD Platform

An MSD (www.mesoscale.com) electrochemiluminescent assay platform was developed to detect circulating autoantibodies. Purified scFv were biotinylated with biotinamidocaproate-NHS (Sigma cat# B2643) at a ratio of 2 biotins : 1 scFv. Purified scFv were also conjugated to the MSD Sulfo-tag (MSD cat# R91AN-1) at a ratio of 18 tags : 1 scFv for 4 hours at room temperature, in the dark. MSD Sulfo-tag conjugated scFv were used to detect autoantibodies captured by biotinylated scFv immobilized onto wells of streptavidin-coated microtiter plates. Unincorporated biotin or Sulfo-tags present in scFv conjugation reactions were removed with desalting columns (Pierce cat# 89883).

A mixture of biotinylated scFv, Sulfo-tag labeled scFv antibody and diluted serum was incubated over night at +4°C. The mixture was transferred to streptavidin-coated plates (MSD cat# L15SA-2)pre-blocked with MSD blocking buffer (MSD cat.# R93AA-2), incubated for 2 hrs at RT on a shaking platform, washed with PBS-T and emptied, after which MSD read buffer (MSD cat.# R92TC-3) was added. An MSD Sector Imager 2400 plate reader was used to quantify electrochemiluminescence assay signals.

### Statistical Method

To assess the primary hypothesis that these 6 selected scFvs autoantibody candidate biomarkers, individually or jointly, have the ability to predict the patient status (NSCLC cancer vs. normal control), we applied logistic regression model. Univariate analysis was applied by only using one of the candidate autoantibody in the logistic model. Multivariable analysis was carried out using backward selection as well as weighted compound score method. The patient classification prediction performance was assessed by a widely used measurement of diagnostic discrimination: receiver operating characteristic (ROC) curves with corresponding area under the curve (AUC). Bootstrapping techniques were used to validate the models and to obtain a bias-corrected estimate of the model’s diagnostic accuracy. In addition, we tested the secondary hypothesis that these 6 scFvs are potential prognostic biomarkers that can predict patients’ survival by applying Cox proportional hazards models. Kaplan-Meier method was also applied to estimate the overall survival time. The results are shown in the results and discussion section.
